# Krise als Dauerzustand. Die Corona-Pandemie in der Geschichte der Gegenwart

**DOI:** 10.1007/s42520-022-00466-3

**Published:** 2022-12-15

**Authors:** Laetitia Lenel

**Affiliations:** Institut für Geschichtswissenschaften, Humboldt-Universität zu Berlin, Berlin, Deutschland

**Keywords:** Zeitgeschichte, Krise, Krisensemantik, Pandemie, Corona, Sideshadowing, Contemporary history, Crisis, Crisis semantics, Pandemic, COVID-19, Sideshadowing

## Abstract

Als empfundener gesellschaftlicher Ausnahmezustand provozierte die Corona-Pandemie frühzeitig eine Vielzahl zeitgenössischer Deutungen. Der Essay setzt sich mit zwei geschichtswissenschaftlichen Abrissen der Pandemie und der gesellschaftlichen Reaktionen darauf auseinander. Neben einem Vergleich beider Bücher fragt der Beitrag auch, wie die Autoren unser Verständnis der Zeit zwischen 2020 und 2021 erhellen, und diskutiert die Herausforderungen und Gefahren einer Krisengeschichtsschreibung. Krise, so argumentiert der Essay, ist mittlerweile fast immer – eine Tatsache, an der die Geschichtswissenschaft nicht ganz unschuldig ist. So produzieren die narrativen Konventionen der Geschichtswissenschaften das Überraschungsmoment, welches das Gefühl des Ausnahmezustands mit hervorbringt. Der Beitrag skizziert mögliche Gegenstrategien, die den Blick auf gegenwärtige und künftige Herausforderungen schärfen könnten.

## Abstract (en)

The essay engages with two historiographical outlines of the COVID-19 pandemic. After a portray and comparison of the two books, the essay explores how they may enhance our understanding of the time between 2020 and 2021 and discusses the challenges and risks of a crisis historiography. The notion of crisis, the essay claims, has become ubiquitous—a development history writing has played a role in. As I argue, the narrative conventions of historical scholarship tend to generate a sense of rupture and discontinuity that has often been perceived as crisis. The article outlines possible counterstrategies that could sharpen our understanding of present and future challenges.

## Einleitung

Krisen sind immer auch Zeiten gesellschaftlicher Selbstbeschreibung. Die Corona-Pandemie stellt dabei keine Ausnahme dar.^1^ Frühzeitig verwiesen Politiker_innen, Journalist_innen und Wissenschaftler_innen auf vergangene Krisen, um daraus Handlungsanleitungen für die Gegenwart abzuleiten. Motiviert durch das Gefühl, in ‚historischen Zeiten‘ zu leben, begannen Zeitgenoss_innen zeitgleich, den möglichen Zäsurcharakter der Pandemie zu diskutieren und ihre Erfahrungen für die Nachwelt zu dokumentieren und zu archivieren.

Mit „Welt im Lockdown. Die globale Krise und ihre Folgen“, dem neuesten Buch des britischen, an der Columbia University lehrenden Wirtschaftshistorikers Adam Tooze^2^, und „Auf Abstand. Eine Gesellschaftsgeschichte der Pandemie“ des deutschen Medizinhistorikers Malte Thießen^3^ liegen nun erste geschichtswissenschaftliche Deutungen der Corona-Krise vor. Beide Bücher richten sich an ein breites Publikum. Während Tooze eine „Interpretation“ der Gegenwart anbieten und damit „eine intellektuelle Wette“ eingehen möchte (S. 33), betont Thießen den Wunsch, Ereignisse und Eindrücke festhalten zu wollen, die sonst in Vergessenheit geraten könnten (S. 12).

1992 hat sich der britische Historiker Eric Hobsbawm mit der Frage auseinandergesetzt, wie eine als historisch empfundene Gegenwart den historischen Blick auf die Vergangenheit verändert.^4^ Angesichts des Zusammenbruchs der Sowjetunion erschien ihm das 20. Jahrhundert plötzlich als ein „kurzes zwanzigstes Jahrhundert“, in dem ein vergleichsweise knappes ‚goldenes‘ Zeitalter von zwei Phasen der Krise eingerahmt wurde.^5^ Vor dem Hintergrund einer nunmehr Jahrzehnte andauernden Beschwörung eines historisch anmutenden Krisen- und Ausnahmezustands wirken sowohl diese Deutung als auch die von Hobsbawm aufgeworfene Frage überholt. Wo permanent der Ausnahmezustand ausgerufen wird, scheint es wichtiger, danach zu fragen, wie die Geschichtswissenschaft den Blick auf einen offensichtlich auf Dauer gestellten Krisenzustand differenzieren kann. Im Folgenden beschränke ich mich deshalb nicht auf Darstellung und Vergleich der Bücher von Adam Tooze und Malte Thießen, sondern frage auch danach, wie sie unser Verständnis der sogenannten Corona-Krise erhellen. Während Tooze die Zeit zwischen 2020 und 2021 als „Polykrise“ historischen Ausmaßes beschreibt, historisiert und relativiert Thießen die zeitgenössische Deutung des Ausnahmezustands. Ausgehend von der Gegenüberstellung beider Bücher ordne ich die zeitgenössische Krisenwahrnehmung historisch ein und arbeite die Herausforderungen und Gefahren einer Krisengeschichtsschreibung heraus. Zwar neigt die Geschichte der Gegenwart durch die eigene Zeitgenossenschaft und die spezifische Quellengrundlage womöglich besonders dazu, das Gefühl zu verstärken, in historischen Zeiten zu leben. Die „Behauptung eines fundamentalen Bruchs und umfassenden Neubeginns“ (Caspar Hirschi) stellt jedoch kein Alleinstellungsmerkmal der Geschichte der Gegenwart dar. Im zweiten Teil meines Essays argumentiere ich, dass die narrativen Konventionen der Geschichtswissenschaft das Gefühl des Ausnahmezustands möglicherweise mit hervorgebracht haben, und beschreibe Strategien, die diesem Eindruck etwas entgegensetzen und unseren Blick auf gegenwärtige und künftige Herausforderungen dadurch schärfen könnten.

## Krisenwahrnehmung und -interpretation bei Adam Tooze

Adam Tooze untersucht in seinem Buch die Monate zwischen dem öffentlichen Eingeständnis des Coronavirus-Ausbruchs durch den chinesischen Staats- und Parteichef Xi Jinping am 20. Januar 2020 und der Amtseinführung Joe Bidens als Präsident der Vereinigten Staaten ein Jahr später. Seiner Darstellung liegt die Annahme zugrunde, dass die Pandemie wirtschaftspolitische Eingriffe beispiellosen Ausmaßes provoziert habe, welche – so der Wirtschaftshistoriker in der Einleitung – die „Fesseln neoliberaler Zurückhaltung gesprengt“ hätten und demnach als „Vorboten eines neuen Regimes jenseits des Neoliberalismus“ erscheinen würden (S. 25). Andererseits hätten diese Eingriffe ‚von oben‘ letztlich dem Imperativ gehorcht, das Finanzsystem zu stabilisieren. Die Reaktion auf die Pandemie sei demnach janusköpfig gewesen. Sie habe ein neues, reformorientiertes Experimentieren hervorgebracht, zugleich aber gesellschaftliche und geopolitische Ungleichheiten reproduziert und verstärkt.

2020 sei es zudem zu einem „neuen Kalten Krieg“ zwischen den Vereinigten Staaten und China gekommen. Tooze betont zwar, dass diese zeitliche Korrelation „bis zu einem gewissen Grad Zufall“ gewesen sei (S. 29). Er glaubt jedoch, dass die amerikanische republikanische Partei und ihre nationalistischen und konservativen Wähler_innen den Aufstieg Chinas vor dem Hintergrund der „allgemeine[n] Krise des Neoliberalismus im Jahr 2020“ als besonders bedrohlich wahrnehmen mussten (S. 30). Dies sei, so der Autor, in den Entwicklungen zwischen November 2020 und Januar 2021 kulminiert, als Donald Trump sich weigerte, die Wahl Bidens anzuerkennen und zusammen mit führenden Persönlichkeiten einen Mob zum Sturm auf das Kapitol ermutigte.

Nach einer ausführlichen Einleitung legt Tooze im ersten Kapitel dar, wie es zu der Pandemie kommen konnte. Mit dem deutschen Soziologen Ulrich Beck spricht er von „organisierter Unverantwortlichkeit“, um den institutionellen Mangel an Vorbereitung zu beschreiben, der sich 2020 offenbart habe (S. 46). Weltweite Landnahme, die industrielle Schweine- und Hühnerzucht, die Herausbildung immer größerer Ballungsräume, die globale Mobilität, der kommerziell motivierte Einsatz von Antibiotika und die Verbreitung von* fake news* über Impfstoffe hätten das Bedrohungspotenzial trotz medizinischer und ökonomischer Fortschritte enorm verstärkt. Demgegenüber stehe – von diesem Vorwurf nimmt der Autor keine Regierung aus – kein angemessenes öffentliches Gesundheitssystem (S. 46). Internationale Organisationen wie die Weltgesundheitsorganisation (WHO) seien dramatisch unterfinanziert und zudem abhängig von den politischen Interessen ihrer Geldgeber. Krankenhäuser wiederum seien seit den 1980er Jahren zu „Experimentierfeldern für modernes Management“ geworden (S. 56). Das Kriterium der Effizienz habe zu einer Reduktion von Überkapazitäten an Betten und essenzieller Ausrüstung geführt. „Jahrzehntelang“, so Toozes Schlussfolgerung, „waren wir in eine immer risikoreichere Zukunft gerast […], ohne ausreichend finanzierte globale Gesundheitsinstitutionen, um den vorhersehbaren Rückschlägen zu begegnen“ (S. 61).

In den nachfolgenden 13 in insgesamt vier Sektionen angeordneten Kapiteln zeichnet der Autor anschließend die Wochen und Monate nach der Entdeckung des neuen Virus in Wuhan bis zur Amtseinführung Joe Bidens nach. Im ersten Teil berichtet Tooze von den unterschiedlichen Reaktionen der chinesischen und westlichen Regierungen auf den Ausbruch des Virus im Kontext wachsender chinesisch-amerikanischer Spannungen. Er erzählt von den umfassenden und raschen Eindämmungsmaßnahmen in China und ihren wirtschaftlichen Folgen sowie der zögerlichen Reaktion des Westens. Selbstgefälligkeit, mangelndes Verständnis der Folgen globaler Vernetzung und ein politisch motivierter Wunsch, die Stimmung an den Finanzmärkten nicht zu trüben, hätten zu einem „historische[n] Versagen“ westlicher Regierungen geführt, das der Kommunistischen Partei Chinas einen „historischen Triumph“ beschert habe (S. 79, 78). Der erste Teil endet mit dem Beginn des weltweiten Lockdowns im März 2020. Dabei beschreibt der Autor, wie sich trotz nationaler Alleingänge und Abschottungstendenzen rasch eine globale Dynamik entwickelt habe.

Der zweite Teil behandelt die durch die Eindämmungsmaßnahmen ausgelöste wirtschaftliche und politische Krise. Tooze, der die in großen Teilen freiwillige Natur des „Shutdowns“ betont, zeichnet nach, wie die Pandemie erst zu einem Angebotsschock und schließlich zu einem Nachfrageschock und einem massiven Einbruch an der Börse führte, und beschreibt die verheerenden, kaskadenartigen Effekte. In drei Kapiteln schildert und bewertet er anschließend die radikalen geld- und fiskalpolitischen Interventionen der Industrienationen, welche die gesellschaftliche Katastrophe zwar erfolgreich abgewendet, zugleich jedoch bestehende Interessen und Ungleichheiten widergespiegelt und reproduziert hätten. Was ein neuer Gesellschaftsvertrag hätte werden können, so das Urteil des Autors, habe sich bei näherem Hinsehen „als ein konfuses und schlecht konzipiertes Monster“ entpuppt (S. 170). Schwellen- und Entwicklungsländer wiederum weiteten ihre Schuldenaufnahme massiv aus, was, so Tooze, nur so lange funktionieren konnte, wie es Liquidität im Überfluss gab (S. 197).

Im dritten Teil schildert er die Auswirkungen der Corona-Pandemie auf die Kräfteverhältnisse in Europa, China und den USA. Er beschreibt, wie die pandemiebedingte Neuverschuldung Italiens, Frankreichs und Spaniens im Juli 2020 trotz nordeuropäischer Widerstände zur Implementierung einer europäischen Gemeinschaftsschuld führte, was Tooze als „Neustart“ für die EU begrüßt (S. 211). Anders als befürchtet, habe die Pandemie Europa dabei nicht von seiner grünen Agenda abgelenkt, was auch an den dramatischen Naturkatastrophen des Jahres 2020 gelegen habe. Stattdessen habe die Corona-Krise Europa mit Xi Jinping einen überraschenden Verbündeten in seinen umweltpolitischen Vorstößen beschert – zumindest dessen Verlautbarungen im September 2020 nach. Und schließlich hätten die ungleichen Auswirkungen der Corona-Krise auch das chinesisch-amerikanische Kräfteverhältnis rascher verschoben als erwartet, worauf die Regierung Donald Trumps mit einem aggressiven Wirtschaftskrieg gegen China reagiert habe. Wie der Streit um Lockerungen der Eindämmungsmaßnahmen und die „Black Lives Matter“-Proteste habe auch die Auseinandersetzung über den Wirtschaftskrieg „die zunehmende Spaltung“ und „Polarisierung“ der amerikanischen Gesellschaft offenbart, die sich mit der amerikanischen Präsidentschaftswahl im November 2020 in die Politik übertragen habe (S. 252).

Der vierte Teil nimmt den globalen Wettlauf um den Impfstoff, die enorme Neuverschuldung von Schwellen- und Entwicklungsländern und die Konjunkturprogramme in den Industrienationen in den Blick. Dieser Abschnitt, bei Tooze „Interregnum“ genannt, könnte auch mit „Verpasste Chancen“ tituliert sein. So betont der Autor zwar die enorme wissenschaftliche Leistung, innerhalb kürzester Zeit einen Impfstoff entwickelt zu haben, bescheinigt den G‑20-Staaten allerdings Versagen bei der Finanzierung eines weltweiten Impfprogramms. Trotz beispielloser Notausgaben sei niemand bereit gewesen, eine schnelle Immunisierung der gesamten Welt zu finanzieren, obwohl das allein aus wirtschaftlichem Eigeninteresse ratsam gewesen wäre. Auch bei der Frage einer finanziellen Entlastung der von der Krise gebeutelten Entwicklungsländer hätten die G‑20-Staaten versagt. Dies sei nicht nur dem Veto der US-amerikanischen Republikaner zuzuschreiben, das die Bemühungen um eine Zuteilung von Sonderziehungsrechten (SZR) gebremst habe, sondern auch dem neuen „Wall Street Consensus“ (Daniela Gabor). Trotz der Konkurrenz mit chinesischen, russischen und türkischen Kreditgebern hätten europäische und amerikanische Staats- und Regierungschefs viel zu wenig öffentliche Mittel für einkommensschwache Kreditnehmer bereitgestellt und sich stattdessen „auf die Magie der Hebelwirkung und der Finanztechnik“ verlassen (S. 296). Dies habe Länder mit mittlerem Einkommen begünstigt und die ärmsten Länder außen vorgelassen. Auch der EU bescheinigt Tooze Versagen angesichts einer als „bescheiden“ eingestuften fiskalischen Reaktion, welche die Kluft zwischen den USA und der Eurozone weiter vergrößert habe: „Was die Fassade europäischer Selbstzufriedenheit bewahrte“, so der Autor, sei lediglich „die Ruhe an den Anleihemärkten“ (S. 317 f.).

Das Buch bietet einen umfassenden Abriss der Pandemie und ihrer gesellschaftlichen, wirtschaftlichen und geopolitischen Auswirkungen zwischen Januar 2020 und Januar 2021. Der Autor beleuchtet eine Vielzahl von Aspekten und geht auf zahlreiche zeitgenössische Debatten ein, wobei er sich auf ein großes Arsenal englisch- und deutschsprachiger Artikel, Stellungnahmen und Blogbeiträge beruft. Dabei gelingt es Tooze, die Offenheit der damaligen Situation herauszustellen und damit den Krisencharakter der Zeit zwischen Januar 2020 und Januar 2021 nachvollziehbar zu machen. Dies ist, wie er in der Einleitung schreibt, eine bewusste Entscheidung: „[A]nstatt vorschnell die Kontinuitäten dieses halben Jahrhunderts der Geschichte skizzieren oder spekulativ in die Zukunft projizieren zu wollen“, verbleibe das Buch soweit möglich „im Augenblick selbst“ (S. 32).

Diese Nähe hat einen Preis. Eine Episode folgt der nächsten, meist umrissen in wenigen, groben Strichen, sodass oft wichtige Aspekte unberücksichtigt bleiben. Die wahnwitzige Niedrigzinspolitik des türkischen Präsidenten, die zu einer Währungs- und Wirtschaftskrise führte, schildert Tooze beispielsweise als „dramatischste[n] Test für Marktmacht und Resilienz“ und skizziert sie auf nicht einmal zwei Seiten als Kräftemessen zwischen „de[m] starke[n] Mann“ Recep Tayyip Erdoğan, den er einen „Spieler“ nennt, und „den Märkten“ (S. 298 f.). Die chinesischen Kredite an Entwicklungs- und Schwellenländer lobt er im Gegensatz zur inadäquaten finanziellen Unterstützung westlicher Staaten als großzügig und visionär und behauptet, der „Geldfluss aus China finanzierte eine beeindruckend moderne Infrastruktur“ (S. 294). Hinweise auf die für Kreditnehmer verheerenden Kreditbedingungen weist er als „Gerüchte“ zurück und schreibt: „Peking zeigte keine Anzeichen dafür, die Corona-Krise auszunutzen, um weiteren Einfluss zu gewinnen“ (S. 287). Auch Pekings Weigerung, sich an der *Debt Service Suspension Initiative* (DSSI) zu beteiligen, will Tooze nicht kommentieren und schreibt lediglich, „Trumps designierter Präsident der Weltbank, David Malpass, machte China dafür verantwortlich, dass die China Development Bank nicht an der DSSI beteiligt war. […] Unterdessen machte Peking bei seinen offiziell anerkannten Krediten gegenüber seinen Schuldnern Zugeständnisse, die so groß waren wie die aller anderen Kreditgeber zusammengenommen“ (S. 287). Ein Beleg fehlt; Differenzierungen sucht man vergeblich. Stimmen, die auf die Intransparenz der chinesischen Kredite und die ungleich höhere Verhandlungsmacht hinweisen, welche die chinesischen Verträge der Gläubigerseite einräumen, bleiben unberücksichtigt; auch die Problematik mangelnder oder laxer chinesischer Vorgaben für Reformen, Wirtschaftlichkeit sowie Sicherheits- und Umweltstandards findet keine Erwähnung.^6^ Damit aber mindert der Autor auch die Überzeugungskraft seiner – an sich richtigen – Forderung nach einer Ausweitung der finanziellen Unterstützung durch westliche Staaten an Entwicklungs- und Schwellenländer.

Etwas mehr Einordnung wäre auch in anderer Hinsicht wünschenswert gewesen. So betont Tooze wiederholt den „einzigartigen“ (S. 9, 189), „beispiellosen“ oder „absolut beispiellosen“ (S. 13, 17, 111, 122, 124, 142, 193, 209, 273, 313, 318, 325), nie dagewesenen (S. 9, 13 f., 77, 124, 148, 170); „undenkbaren“ (S. 22) und „historischen“ Charakter (u. a. S. 11, 78 f., 132, 136, 151, 162) der von ihm beschriebenen Entwicklungen (im englischen Original werden hier meist die Begriffe „unprecedented“ und „historic“/„historical“ verwendet). Die Welt, lernt die Leserin, „taumelte“ (S. 13); Europa geriet „ins Wanken“ (S. 126). Tatsächlich fühlte sich das 2020 mitunter so an. Bezeichnenderweise nimmt der Autor immer wieder Anleihen bei den Deutungen zeitgenössischer Akteure. So übernimmt er den von dem ehemaligen EU-Kommissionspräsidenten Jean-Claude Juncker populär gemachten Begriff der „Polykrise“ und den auf den chinesischen Politiker Chen Yixin zurückgehenden Ausdruck der konvergierenden Krisen (vgl. dazu vor allem S. 14–16, aber auch S. 97, 208, 314, 343). Zwar vermag diese Sprache etwas von der empfundenen Dramatik und Atemlosigkeit der damaligen Situation zu vermitteln. Als historisches Deutungsangebot lässt es die Leserin jedoch ratlos zurück. Taumeln wir seitdem? Taumeln wir seit 2010, dem Jahr, das für Juncker den Beginn der Polykrise bildete? Oder gar seit 1993, als Edgar Morin und Anne Brigitte Kern den Begriff der Polykrise prägten? Welchen analytischen, aber auch politischen Mehrwert haben Krisenbegriff und -semantik, wenn die Krise einen Dauerzustand beschreibt?

## Die Pandemie und ihre Vorläufer bei Malte Thießen

Malte Thießens Buch kann diese Ratlosigkeit ein Stück weit auflösen. Seine „Gesellschaftsgeschichte der Pandemie“ bietet in zehn kurzen Kapiteln einen Abriss der bundesrepublikanischen Reaktion auf die Pandemie 2020/21. Durch zahlreiche Verweise auf frühere Pandemien und ihre gesellschaftlichen Folgen kann der Medizinhistoriker verdeutlichen, dass die Reaktionen auf die Corona-Pandemie mitunter lange Traditionen hatten. In historischer Perspektive, so Thießen, erscheinen Reaktionen vom *Othering* und der Stigmatisierungen einzelner Gesellschaftsgruppen bis zur gesellschaftlichen Aushandlung der Balance zwischen freiem Handel und Sicherheit „erstaunlich bekannt bzw. erschreckend vertraut“ (S. 11). Thießen zeichnet diese Parallelen detailliert nach. So verdeutlicht er beispielsweise in einem aufschlussreichen Kapitel über die Proteste von Imfgegner_innen, dass sich nicht nur Impfkritik und -proteste bis ins 19. und frühe 20. Jahrhundert zurückverfolgen lassen, sondern dass auch die Internationalität heutiger Proteste, die „Echokammern des Internets“ und die geschichtspolitische Konnotation von Gesundheitsthemen jahrzehntealte historische Vorläufer haben (S. 129–134).

Zugleich verliert Thießen über diese Gemeinsamkeiten nicht die unterschiedlichen Kontexte verschiedener Pandemien aus dem Blick, denen er die verschiedenen gesellschaftlichen Reaktionen zuschreibt. So erklärt er sowohl das zögerliche Handeln der deutschen Regierung im Frühjahr 2020 als auch die anschließend unternommenen tiefgreifenden Eindämmungsmaßnahmen mit dem spezifischen Erfahrungsraum der deutschen Gesellschaft im Jahr 2020. Die zögerliche Reaktion Anfang 2020 führt der Autor etwa auf die Erfahrungen mit der Schweinegrippe im Herbst 2009 zurück. Schließlich sei die damalige entschlossene Seuchenbekämpfung anschließend als „teure Panikmache“ gebrandmarkt worden, was Gesundheitspolitiker_innen 2020 „den hohen Schädigungsfaktor einer übertriebenen Seuchenbekämpfung schmerzlich bewusst“ gemacht habe (S. 24). Thießen hebt aber auch die Bedeutung der medizinischen Erfolgsgeschichte seit den 1970er Jahren hervor. Diese habe dazu geführt, dass Seuchen – anders als noch in den 1960er Jahren – keine Alltagserfahrungen mehr darstellten, was Anfang 2020 ein trügerisches und lähmendes Gefühl der Sicherheit erzeugt habe. Auch die Deutung der Pandemie als „Ausnahmezustand“ versteht der Autor in diesem Kontext, wobei er in einem aufschlussreichen Abriss zudem herausarbeitet, inwiefern die Rede vom Ausnahmezustand im gesamten Parteienspektrum zum schlagkräftigen Argument instrumentalisiert wurde (S. 69–75). Die – im Vergleich zu früheren Pandemien – sehr viel umfassenderen Eindämmungsmaßnahmen wiederum begründet Thießen unter anderem damit, dass sich seit den 1970er Jahren neue Erwartungen an das „Vierte Lebensalter“ durchgesetzt hätten. Dadurch sei das Leben von Alten und Vorerkrankten „in einem ganz anderen Maße als lebens- und schützenswert“ bewertet worden als noch fünf Jahrzehnte zuvor (S. 51). Die Reaktion der Grenzschließungen sei hingegen auch im Kontext einer Pathologisierung der Globalisierung zu lesen, die seit der zweiten Hälfte des 20. Jahrhunderts an Fahrt gewonnen habe. Mit diesen „unterschiedlichen Erfahrungsräumen“ (S. 48) kann der Autor die politischen Reaktionen auf die Corona-Pandemie und die Unterschiede zu den Antworten auf frühere Seuchen erklären, darunter beispielsweise die „Hongkong-Grippe“ der späten 1960er Jahre.

Thießen bezeichnet die Pandemie auch als „Seismograph des Sozialen, der die Tektonik der deutschen Gesellschaft und deren soziale Verwerfungen“ offenlege (S. 75). Er nimmt entsprechend nicht nur die Wirkungen der Pandemie auf die soziale Ungleichheit in den Blick, sondern schildert auch verschiedene gesellschaftliche Debatten und ihren Wandel, wobei das behandelte Spektrum von den Diskussionen über das Maskentragen und die Impfung bis zur Debatte über das Verhältnis zwischen Wissenschaft und Politik reicht. Hier wirkt die Darstellung mitunter etwas disparat, gewinnt durch unerwartete historische Vergleiche aber immer wieder an Tiefenschärfe. Hinweise auf den internationalen Kontext und globale Dynamiken fehlen; eine Leerstelle, die das Buch von Adam Tooze wiederum füllt.

Wo Tooze den atemlosen Ton der Zeitgenossen übernimmt und die Dramatik der Situation durch eine mitunter recht unvermittelt wirkende Aneinanderreihung von Einzelepisoden zusätzlich hervorhebt, relativiert Thießen die zeitgenössische Deutung der Pandemie als „Ausnahmezustand“. Als eigentliche „seuchengeschichtliche Zäsur“ bewertet er die Tatsache, dass keine Pandemie zuvor „auch nur in Ansätzen so gut dokumentiert worden [sei] wie die von 2020/21“ (S. 146). Auf der Grundlage dieser zeitgenössischen Quellen gelingt es dem Autor, die Corona-Krise als Wahrnehmungsphänomen greifbar zu machen – ein Schritt, der angesichts der zahlreichen Diskussionen über den Zäsurcharakter der Pandemie besonders vielversprechend scheint.^7^ Das Zeitempfinden an sich, so Thießen, belege noch keine Zäsur. „Menschen tendieren in Krisen schnell dazu, Ereignissen einen Zäsurcharakter zuzuschreiben. Meist lösen sich solche Zuschreibungen jedoch im Strom der Geschichte auf“ (S. 179).

## Krise als Dauerzustand und die Rolle der Geschichtsschreibung

Warum aber scheinen die Zeitenwenden einander aktuell geradezu zu jagen? Von Frank-Walter Steinmeiers Aussage aus dem April 2020, die Welt danach werde eine andere sein (S. 179), liest man 2022 nicht ohne gewisses Erstaunen: Hat Olaf Scholz die „Zeitenwende“ nicht erst im Februar dieses Jahres ausgerufen? Auch mit Blick auf die Zeit vor 2020 scheint die Beschwörung der Zäsur beziehungsweise eines „die Welt verändernden“ Krisenzustands allgegenwärtig. So berichtet Tooze davon, wie er sich nach der Veröffentlichung seines Buchs „Crashed. Wie zehn Jahre Finanzkrise die Welt verändert haben“ im Jahr 2019 eigentlich der weiter zurückliegenden Vergangenheit zuwenden wollte. Die Krisen der Gegenwart hätten ihn jedoch zur „Kapitulation“ gezwungen: „Ich hatte mir vorgenommen, ein Buch zum zehnten Jahrestag der Finanzkrise zu schreiben, und landete nach dem Brexit und Trumps Sieg mitten in einer Krise, die nicht enden wollte“ (S. 33).

Immer wieder hoben Zeitgenoss_innen zuletzt den neuartigen Charakter des Gefühls der Krise und der Zeitenwende hervor. So argumentierte beispielsweise der Soziologe Andreas Reckwitz kürzlich, dass es in Westeuropa und Nordamerika nach 1945 und insbesondere seit 1990 zu einem „Revival“ des Fortschrittsdenkens gekommen sei, das erst mit der russischen Invasion der Ukraine einen jähen „Schock“ erlitten habe.^8^ Der Angriffskrieg habe dem „Glauben an die Unvermeidlichkeit einer historischen Entwicklung in jene Richtung, die man als Fortschritt erkannt hat“, ein plötzliches Ende gesetzt. Von dieser Auffassung zeugen auch andere Artikel der letzten Monate. So erklärte beispielsweise Elena Witzeck im Juli 2022 in der „Frankfurter Allgemeinen Zeitung“, dass die Generation der heute Mittdreißiger noch an „tausend Zukünfte“ geglaubt habe – und an „die Unerschöpflichkeit der Ressourcen“.^9^ Sie sei – hier zitierte die Autorin Frederic Hanusch, der kürzlich mit Claus Leggewie und Erik Meyer ein Buch über das „Planetare Denken“ veröffentlicht hat – mit einem „linearen Fortschrittsgedanken“ aufgewachsen.^10^

Zeitgenössische Deutungen entlarven Interpretationen wie diese als retrospektive Verklärung. Eine „Zukunft ohne Illusion“, wie Witzeck sie fordert, wird seit Jahrzehnten ausgerufen. So datierte beispielsweise der Politologe Wolf Schäfer Mitte der 1980er Jahre den „Anfang vom Ende der positiven Version des Fortschritts“ im *fin de siècle*, von dem er einen Bogen von Karl Kraus in den 1920er Jahren über die dystopische Science-Fiction-Literatur der späten 1940er Jahre bis zur eigenen Gegenwart spannte: „Die gegenwärtige Version des Fortschritts ist von einer historisch gewachsenen Desillusionierung geprägt.“^11^ Es sei, so Schäfer, unbestreitbar, dass „das Projekt der Moderne aus dem Ruder läuft und immer tiefer in politische Klippen, ökologische Strudel und demographische Engpässe steuert“.^12^ Tatsächlich waren Wahrnehmungen wie diese spätestens in den 1970er Jahren zum Allgemeinplatz geworden – und blieben es auch in den darauffolgenden Jahren. Zeitgenössische Deutungen der 1980er und 1990er Jahren kreisten um die „Grenzen des Wachstums“ und thematisierten eine grassierende Zukunftsangst.^13^ Auch die oft zitierte Rede vom „Ende der Geschichte“ der angeblich so fortschrittsgläubigen 1990er Jahre wurde bereits wenige Jahre später als Ergebnis „erste[r] euphorische[r] Momente“ verworfen. „Das Ende der Geschichte sah rasch aus wie die trübe Auferstehung halbtoter Affekte aus dem 19. Jahrhundert,“ erklärte beispielsweise Gustav Seibt 1999.^14^ Stattdessen machten Deutungen wie jene der „Polykrise“ die Runde, mit der Edgar Morin und Brigitte Kern 1993 die komplexe Gemengelage aus Krisen, unkontrollierbaren Prozessen und der allgemeinen Krise des Planeten bezeichneten.^15^ 2002 forderte der französische Philosoph Jean-Pierre Dupuy angesichts der Krisen, Risiken und menschlichen Selbstzerstörung einen „aufgeklärten Katastrophismus“, der sich den Gefahren stelle, ohne die Hoffnung zu verlieren, „dass diese Zukunft, obwohl sie unausweichlich ist, nicht stattfindet.“^16^ Von einem linearen Fortschrittsgedanken ist hier wenig zu finden.

Der amerikanische Anthropologe Joseph Masco hat die Häufigkeit eines politisch und medial beschworenen Krisenzustands 2017 als „überwältigend“ beschrieben und von einem „sich ständig verstärkenden Medienrefrain“ gesprochen.^17^ Masco argumentierte, dass der Begriff der Krise in Form eines „ever-present, near permanent negative ‚surround‘“ unscharf gemacht worden sei und darüber seine mobilisierende Kraft eingebüßt habe.^18^ So provoziere die ständige Ausrufung des Krisenzustands kurzfristige Lösungsansätze und lenke darüber von den eigentlich notwendigen Systemveränderungen ab. Der Begriff der Krise sei dadurch zu einem „counterrevolutionary idiom“ avanciert, dessen Verwendung inzwischen der Stabilisierung des Status Quo diene: „Crisis talk today seeks to stabilize an institution, practice, or reality rather than interrogate the historical conditions of possibility for that endangerment to occur.“^19^

Könnte es sein, dass Historiker_innen an der ständigen Beschwörung des Krisenzustands nicht ganz unschuldig sind oder diesem Trend zumindest nicht genug entgegensetzen? Der Schweizer Historiker Caspar Hirschi attestierte den populären geschichtswissenschaftlichen Jahreszahl-Büchern kürzlich einen Hang zur „Zweiteilung der Geschichte in eine Zeit vor und nach der betreffenden Jahreszahl, verbunden mit der Behauptung eines fundamentalen Bruchs und umfassenden Neubeginns, der eine direkte Kontinuität bis zum Hier und Heute einleitet“.^20^ Hirschis Beobachtung ließe sich leicht verallgemeinern. Auch andere historische Darstellungen stellen ihre eigene Relevanz mit dem Hinweis heraus, die entscheidende Krise zu behandeln, die zugleich einen Schlüssel für die eigene Gegenwart biete.

Geschichten der Gegenwart können diese Tendenz zusätzlich verstärken. Thießen hebt den Zugang zur digitalen Überlieferung als eines „der größten Potenziale“ der Zeitgeschichte hervor, würden Stellungnahmen auf Blogs, Tweets, Bilder und Fotos doch einerseits „neue Einblicke in eine Pandemie“ eröffnen und seien andererseits besonders bedroht, weil viele digitale Quellen in den kommenden Jahren verloren gehen könnten (S. 12). Diese Quellengrundlage macht einen großen Reiz beider Bücher aus, ermöglicht sie den Autoren doch auch, erstaunlich vielfältige Perspektiven zur Sprache zu bringen. Werden diese Deutungen nicht eingeordnet und kontextualisiert, birgt die Gegenwartsnähe jedoch die Gefahr, das zeitgenössische Gefühl, in ‚historischen Zeiten‘ zu leben, noch zu verstärken. Das gilt ganz besonders, wenn Tooze Anleihen bei den Begriffen und Deutungen der Zeitgenossen nimmt, aber auch, wenn Thießen das „Wir“ der zeitgenössischen Beobachter_innen verwendet. So distanziert sich Thießen im Ausblick zwar von zeitgenössischen Deutungen der Pandemie als „Zeitenwende“ (S. 179), schreibt auf der ersten Seite seines Buchs aber selbst: „Der Lockdown riss uns aus dem gewohnten Leben und markierte den Beginn einer neuen Zeitrechnung: der Zeit vor und nach Corona“ (S. 9). Wie Tooze, der an einer Stelle Emmanuel Macrons Begriff des „tiefgreifenden anthropologischen Schocks“ zitiert, um die globalen Dynamiken des Shutdowns zu beschreiben (S. 100), scheint Thießen hier nicht nur eine zeitgenössische Wahrnehmung wiederzugeben, sondern sich diese auch zu eigen zu machen. Der Fokus auf Geschichte, die „noch qualmt“, so könnte man in Anlehnung an Barbara Tuchman sagen, begünstigt möglicherweise die Vermittlung – und mitunter Verstärkung – der „*Stimmung* einer Episode oder eines geschichtlichen Augenblicks [Hervorh. im. Orig.]“.^21^

Ist dieser Schulterschluss zwischen den Historiker_innen und den Subjekten ihrer Forschung nur der Zeitgenossenschaft und der spezifischen Quellengrundlage sowie, im Fall der allgemeinen Geschichtswissenschaft, dramaturgischen Überlegungen und Verlagskalkül geschuldet? Oder könnten die Konventionen narrativer Geschichtswissenschaft die Wahrnehmung und Stilisierung der erzählten Zeit als Zeitenwende zusätzlich begünstigen?

Einer der berühmtesten Thesen Reinhart Kosellecks zufolge kam es im Zuge der Französischen Revolution und den damit verbundenen Umwälzungen zu einer „Verzeitlichung der Geschichte“.^22^ Während sich die Menschen bis zum späten 18. Jahrhundert in einem Geschichtskontinuum gewähnt hätten, in dem vergangene Erfahrungen als exemplarisch und handlungsleitend für Gegenwart und Zukunft angesehen worden seien, habe die Geschichte seit Anbruch der Moderne ihren Stellenwert als Lehrmeisterin des Lebens eingebüßt.^23^ Die Erfahrung von Wandel und Beschleunigung habe den Glauben an die potenzielle Gleichförmigkeit und Wiederholbarkeit von Geschichte ausgehöhlt. In der Folge seien der „Erfahrungsraum“ und der „Erwartungshorizont“ der Zeitgenossen auseinandergetreten.^24^ Die moderne Geschichtsschreibung, so Koselleck, sei letztlich ein Produkt der Einsicht in die grundsätzliche Differenz zwischen Vergangenheit und Gegenwart.

Kosellecks These einer Verzeitlichung der Geschichte um 1800 wurde von der Forschung schon mehrfach zurückgewiesen. Einerseits haben Historiker_innen der Frühen Neuzeit darauf hingewiesen, dass Zeitgenossen auch vor Anbruch der Moderne nicht von einer grundsätzlichen Gleichförmigkeit der Geschichte ausgegangen seien. Dies würde beispielsweise die Heiratspolitik hochadliger Familien belegen, die von einem starken Vertrauen in die Gestaltbarkeit der Zukunft zeuge.^25^ Auch hätten Zeitgenoss_innen schon in der Frühen Neuzeit über einen ausgeprägten Zukunftsbezug verfügt, was sich unter anderem in der zeitgenössischen Literatur, aber auch in dem prominenten Stellenwert verschiedener Prognosepraktiken manifestiert habe.^26^ Andererseits haben Historiker_innen der Neuesten Geschichte aufgezeigt, dass sich der Topos der Geschichte als Lehrmeisterin mit Anbruch der Moderne keineswegs aufgelöst habe, wie von Koselleck behauptet. Im Gegenteil habe ein Großteil der Geschichtsschreibung diesen Anspruch auch im 19. Jahrhundert und darüber hinaus propagiert.^27^ Mehrere Forscher haben zudem dafür plädiert, die „Verzeitlichung der Geschichte“ auf das 17. Jahrhundert vorzudatieren.^28^

Die von Koselleck postulierte Kausalität zwischen dem Aufkommen eines neuen Zeitgefühls und der Entstehung der modernen Geschichtsschreibung wurde dabei bislang nicht infrage gestellt. Es spricht jedoch viel dafür, die Geschichtsschreibung selbst als Faktor einer Veränderung des Zeitgefühls ernst zu nehmen und – anders als Koselleck – von einer reziproken Beeinflussung von Zeitgefühl und moderner Geschichtsschreibung auszugehen. So setzte um die Mitte des 16. Jahrhunderts ein lebhafter Diskurs über die Grenzen und Ziele der Geschichtsschreibung ein, infolgedessen sich neue narrative Konventionen herausbildeten, welche die Erfahrung geschichtlichen Wandels begünstigten und zum Teil auch ermöglichten. Autoren der *ars historica*-Tradition wie die französischen Juristen François Baudouin und Jean Bodin postulierten die Notwendigkeit einer profunden Quellenkritik und reflektierten die Standortgebundenheit und Perspektivität von Geschichte.^29^ Befeuert wurden diese theoretischen Debatten durch die Aufdeckung zahlreicher mittelalterlicher und frühneuzeitlicher Fälschungen antiker Quellen im 15. und 16. Jahrhundert, die neues Augenmerk auf die Historizität von Quellen und der Geschichtsschreibung lenkte.^30^ In der Folge bildete die Geschichtsschreibung bereits im späten 16. Jahrhundert neue Konventionen einer narrativen oder prozessualen Geschichtsschreibung heraus, die historische Ereignisse nicht mehr als bloße chronologische Abläufe darstellte, sondern verschiedene Ereignisse in einen kausalen Zusammenhang brachte. Wo der Standort der Quellen ein so grundsätzlich anderer war als der Standort der Historiker, dass er erst vor dem Hintergrund ihres Entstehungskontextes entziffert werden konnte, wurde die Diskrepanz zwischen erzählter Zeit und Erzählzeit selbst erklärungsbedürftig. In der Folge florierte eine prozessuale Geschichtsschreibung, die versuchte, durch narrative Verknüpfungen die Entwicklungen und Kontinuitäten nachzuzeichnen, welche die erzählte Zeit mit der Erzählzeit verbanden – und auf die Zukunft der Leser_innen bezog.^31^

Diese Konventionen sind bis heute gültig geblieben und reproduzieren das Überraschungsmoment, das ein Gefühl permanenten Wandels erzeugt und Kosellecks These dadurch intuitiv zu bestätigen scheint. Wo Ereignisse nachträglich in einen kausalen Zusammenhang gebracht werden, erscheinen vermeintlich gegenläufige Tendenzen als „Bruch“ oder „Wende“. In einem Artikel über die wissenschaftliche Prognostik und Antizipation sprach der britische Philosoph Mark Currie von der „heightened experience of surprise produced by the labor of its annihilation.“^32^ Die Vorstellung, dass die Welt weniger vorhersehbar sei als früher, müsse demnach als Folge der Prognostik, nicht als Ausdruck ihres Scheiterns verstanden werden. Ebenso könnte man auch den Eindruck, Zeugin einer Zeitenwende zu sein, als Produkt der retrospektiven Kohärenzstiftung interpretieren. Schließlich begünstigt die Erwartung grundsätzlicher Kontinuität das Gefühl, in historischen, nie dagewesenen Zeiten zu leben.

Koselleck hob die Bedeutung der „vehemente[n] Erfahrung der Französischen Revolution“ hervor, „die alle bisherigen Erfahrungen zu überholen schien“, und damit die Entstehung der modernen Geschichtsschreibung ermöglicht habe.^33^ Die moderne Geschichtsschreibung produziert die Erfahrung der Krise und Zeitenwende jedoch auch – und zwar ständig aufs Neue.

## Fazit

In seiner Einleitung schreibt Tooze: „Das Jahr 2020 entpuppte sich als wahrhaft historisch, als völlig anders als alles, was wir je zuvor erlebt hatten“ (S. 33). Das ist richtig und wurde sicherlich von vielen Zeitgenoss_innen so empfunden, könnte aber auch über jedes andere Jahr gesagt werden. Wie entkommen wir der Gefahr einer Steigerungslogik, in der jedes neue Jahr als krisenhafter und historischer als das letzte dargestellt wird?

In zwei wegweisenden Büchern plädierten die Literaturwissenschaftler Gary Saul Morson und Michael André Bernstein 1994 dafür, die Offenheit vergangener Gegenwarten greifbar zu machen.^34^ Statt retrospektiv eine angeblich notwendige Kausalität zwischen verschiedenen Vergangenheiten zu postulieren, die in unserer eigenen Gegenwart kulminiert, schlugen sie eine Praxis des „Sideshadowing“ vor, die den Möglichkeitsraum vergangener Gegenwarten greifbar macht: „[T]he temporal world consists not just of actualities and impossibilities but also of real though unactualized possibilities. Sideshadowing invites us into a peculiar *middle realm* [Hervorh. im Orig.].“^35^ Dadurch werde nicht nur begreiflich, dass die Gegenwart nicht das einzig mögliche Ergebnis früherer Zeiten sei, sondern auch, dass es verschiedene mögliche Zukünfte gebe. Morsons und Bernsteins Forderung hatte auch eine ethische Dimension: Schließlich sollte die alternative narrative Form im Gegensatz zu den kritisierten Konventionen des *Foreshadowing* und *Backshadowing* auch individuelle Wahlfreiheit und Verantwortung demonstrieren. Als Gegenmittel zur retrospektiven Kohärenzstiftung könnte die Praxis des *Sideshadowing* aber auch ein Mittel sein, die Erfahrung des Krisen- und Ausnahmezustands zu relativieren.

Für die Geschichte der Gegenwart würde das neben dem Überdenken des analytischen Mehrwerts des Krisenbegriffs auch bedeuten, größere Vorsicht bei der Projektion der Gegenwart in die Zukunft walten zu lassen. „‚We ain’t seen nothing yet.‘ Was wir bisher erlebt haben, das war erst der Anfang“, lautet der letzte Satz von Toozes Darstellung (S. 343). Vor dem Hintergrund der Krisendynamiken, die er beschreibt, muss dieser Satz bedrohlich wirken. Tatsächlich meint Tooze, dass wir uns in einem „Prozess der Eskalation“ befinden und dass „eine Deeskalation schwer, wenn nicht gar unvorstellbar“ sei (S. 338 f.). Joseph Masco nannte den Fokus auf die „endless modes of precarity that are emerging“ einen „perverse effect“ des permanenten Krisendiskurses.^36^ Er hielt es für zentral, die Fähigkeit wiederzuerlangen, positive Zukunftsentwürfe zu generieren, die kollektives Handeln motivieren. Überzeugt, dass Angst eher lähmt als ermutigt, erklärte die kanadische Klimaforscherin Corinne Le Quéré 2018 in einem TEDx-Talk: „Before I die, we will recylce everything […] we will no longer eat animals […] we will breathe pure air in the heart of our cities.“^37^ Und warum sollte die Zukunft nicht derart historisch werden?

## Besprochene Literatur

- Thießen, Malte: Auf Abstand. Eine Gesellschaftsgeschichte der Coronapandemie, 222 Seiten, Campus, Frankfurt a. M. 2021.
- Tooze, Adam: Shutdown. How Covid Shook the World’s Economy, 368 Seiten, Allen Lane, London 2021.
- Tooze, Adam: Welt im Lockdown. Die globale Krise und ihre Folgen, übers. v. Andreas Wirthensohn, 408 Seiten, Beck, München 2021.

## Footnotes

1 Einen guten Überblick zur deutschen Reaktion auf die Corona-Pandemie bieten Lehmann, Benet/Schacher, Paul: Geschichtskultur in der Corona-Pandemie. Beobachtungen zur Historisierung einer aktuellen Krise, in: Beuerbach, Jan u. a. (Hrsg.): Covid-19. Sinn in der Krise. Kulturwissenschaftliche Analysen der Corona-Pandemie, De Gruyter, Berlin 2021, S. 337–356.

2 Tooze, Adam: Welt im Lockdown. Die globale Krise und ihre Folgen, übers. v. Andreas Wirthensohn, Beck, München 2021 (engl. 2021).

3 Thießen, Malte: Auf Abstand. Eine Gesellschaftsgeschichte der Coronapandemie, Campus, Frankfurt a. M. 2021.

4 Hobsbawm, Eric: The Present as History. Writing the History of One’s Own Time, in: Bates, David/Wallis, Jennifer/Winters, Jane (Hrsg.): The Creighton Century, 1907–2007, UoL Press, London 2020, S. 269–285; siehe dazu Lenel, Laetitia: Geschichte ohne Libretto, in: H‑Soz-Kult. Forum: Zeiterfahrung, 05. Dezember 2020, URL: <www.hsozkult.de/debate/id/diskussionen-5090> [Zugriff: 31.08.2022].

5 Hobsbawm: Present (wie Anm. 4), S. 281.

6 Vgl. beispielsweise die auf der Seite des Kiel Instituts für Weltwirtschaft zu findende Studie von Gelpern, Anna u. a.: How China Lends. A Rare Look into 100 Debt Contracts with Foreign Governments. March 2021, URL: <https://www.ifw-kiel.de/de/publikationen/journal-article/2021/how-china-lends-a-rare-look-into-100-debt-contracts-with-foreign-government-16100/> [Zugriff: 31.08.2022].

7 Mergel, Thomas: Einleitung. Krisen als Wahrnehmungsphänomene, in: ders. (Hrsg.): Krisen verstehen. Historische und kulturwissenschaftliche Annäherungen, Campus, Frankfurt a. M. 2012, S. 9–22.

8 Reckwitz, Andreas: Der Optimismus verbrennt, in: Die Zeit, Nr. 12, 19. März 2022.

9 Witzeck, Elena: Zukunft ohne Illusion, in: Frankfurter Allgemeine Zeitung, 29. Juli 2022, S. 9.

10 Hanusch, Frederic/Leggewie, Claus/Meyer, Erik: Planetar denken. Ein Einstieg, transcript, Bielefeld 2021.

11 Schäfer, Wolf: Die Krankheit der Vernunft, in: Die Zeit, Nr. 15, 03. April 1987; vgl. auch ders.: Die unvertraute Moderne. Historische Umrisse einer anderen Natur- und Sozialgeschichte, Fischer Taschenbuch, Frankfurt a. M. 1985.

12 Ders.: Moderne (wie Anm. 11), S. 12.

13 Siehe beispielsweise Seibt, Gustav: Endliche Welt, kein Ende der Geschichte, in: Gall, Lothar (Hrsg.): Das Jahrtausend im Spiegel der Jahrhundertwenden, Propyläen, Berlin 1999, S. 379–418, hier S. 400.

14 Ebd., S. 411.

15 Morin, Edgar/Kern, Anne Brigitte: Homeland Earth. A Manifesto for the New Millenium. Advances in Systems Theory, Complexity, and the Human Sciences, Hampton Press, Cresskill, NJ 1999, S. 74; das französische Original erschien 1993 als dies.: Terre-Patrie, Seuil, Paris 1993.

16 Dupuy, Jean-Pierre: Pour un catastrophisme éclairé. Quand l’impossible est certain, Seuil, Paris 2004, S. 216.

17 Masco, Joseph: The Crisis in Crisis, in: Current Anthropology 58, Nr. S15 (2017), H. 2, S. S65–S76, hier S. S65; vgl. dazu auch Lenel: Geschichte (wie Anm. 4).

18 Masco: Crisis (wie Anm. 17), S. S65.

19 Ebd., S. S73.

20 Hirschi, Caspar: Wie Science-Fiction Geschichte wird – und umgekehrt. Zu den Verwandlungen des Jahreszahl-Buches, in: Mittelweg 36, Jg. 31 (2022), H. 2, S. 70–83, hier S. 75.

21 Tuchman, Barbara Wertheim: Wann ereignet sich Geschichte?, in: dies.: In Geschichte denken. Essays, übers. v. Rudolf Schultz und Eugen Schwarz, Fischer Taschenbuch, Frankfurt a. M. 1984 (engl. 1982), S. 31–39, hier S. 31, 39.

22 Koselleck, Reinhart: Vergangene Zukunft der frühen Neuzeit (1979), in: ders.: Vergangene Zukunft. Zur Semantik geschichtlicher Zeiten, Suhrkamp, Frankfurt a. M. 1989, S. 17–37, hier S. 19.

23 Ders.: Historia Magistra Vitae. Über die Auflösung des Topos im Horizont neuzeitlich bewegter Geschichte (1979), in: ders.: Zukunft (wie Anm. 22), S. 38–66.

24 Ders.: ‚Erfahrungsraum‘ und ‚Erwartungshorizont‘ – zwei historische Kategorien (1979), in: ders.: Zukunft (wie Anm. 22), S. 349–375.

25 Burke, Peter: Foreword. The History of the Future, 1350–2000, in: Brady, Andrea/Butterworth, Emily (Hrsg.): The Uses of the Future in Early Modern Europe, Routledge, London/New York 2010, S. ix–xx, hier S. xv.

26 Franklin, James: The Science of Conjecture. Evidence and Probability before Pascal, JHUP, Baltimore, MD 2001; Burke: Foreword (wie Anm. 25); Barret, J. K: Untold Futures. Time and Literary Culture in Renaissance England, Cornell UP, Ithaca, NY 2016; Deringer, William: Calculated Values. Finance, Politics, and the Quantitative Age, Harvard UP, Cambridge, MA/London 2018; ders.: Compound Interest Corrected. The Imaginative Mathematics of the Financial Future in Early Modern England, in: Osiris 33 (2018), H. 1, S. 109–129.

27 Bouton, Christophe: Learning from History. The Transformations of the Topos *Historia Magistra Vitae* in Modernity, in: Journal of the Philosophy of History 13 (2019), H. 2, S. 183–215.

28 Vgl. Meumann, Markus: Von der Endzeit zum Säkulum. Zur Neuordnung von Zeithorizonten und Zukunftserwartungen ausgangs des 17. Jahrhunderts, in: Heudecker, Sylvia/Niefanger, Dirk/Wesche, Jörg (Hrsg.): Kulturelle Orientierung um 1700. Traditionen, Programme, konzeptionelle Vielfalt, Niemeyer, Tübingen 2004, S. 100–121; Fulda, Daniel: Wann begann die ‚offene Zukunft‘? Ein Versuch, die Koselleck’sche Fixierung auf die Sattelzeit zu lösen, in: Breul, Wolfgang/Schnurr, Jan Carsten (Hrsg.): Geschichtsbewusstsein und Zukunftserwartung in Pietismus und Erweckungsbewegung, Vandenhoeck & Ruprecht, Göttingen 2013, S. 141–172.

29 Vgl. Wolf, Johannes: Io. Bodini Methodus historica: duodecim eiusdem argumenti scriptorum, tarn veterum quam recentiorum, quorum elenchum praefationi subiecimus, Perna, Basel 1576, S. 646–647; Bodin, Jean: Method for the Easy Comprehension of History, übers. v. Beatrice Reynolds, Norton, New York 1985, S. 9, 45–55, 336–337; zur Bedeutung der *ars historica*-Tradition vgl. insbesondere Grafton, Anthony: What was History? The Art of History in Early Modern Europe, Cambridge UP, Cambridge 2007.

30 Siehe dazu auch Grafton, Anthony: Defenders of the Text. The Traditions of Scholarship in an Age of Science, 1450–1800, Harvard UP, Cambridge, MA/London 1991, S. 78; vgl. in diesem Zusammenhang auch Lyon, Gregory B.: Baudouin, Flacius, and the Plan for the Magdeburg Centuries, in: Journal of the History of Ideas 64 (2003), H. 2, S. 253–272, hier S. 266 f.

31 Baudouin, François: De institutione historiae universae et eius cum iurisprudentia coniunctione prolegomenon, Paris 1561, S. 9; vgl. dazu auch Kelley, Donald R.: Historia Integra. François Baudouin and his Conception of History, in: Journal of the History of Ideas 25 (1964), H. 1, S. 35–57, hier S. 51–52; Bodin, Jean: Methodus ad facilem historiarum cognitionem, Martin le Jeune, Paris 1572, S. 18 f., siehe auch 31.

32 Currie, Mark: Anticipation/Unexpected, in: Burges, Joel/Elias, Amy J. (Hrsg.): Time. A Vocabulary of the Present, NYU Press, New York 2016, S. 97–110, hier S. 100.

33 Koselleck: Historia (wie Anm. 23), S. 59.

34 Morson, Gary Saul: Narrative and Freedom. The Shadows of Time, Yale UP, New Haven, CT/London 1994; Bernstein, Michael André: Foregone Conclusions. Against Apocalyptic History, California UP, Berkeley, CA u. a. 1994.

35 Morson: Narrative (wie Anm. 34), S. 119.

36 Masco: Crisis (wie Anm. 17), S. S75.

37 Mance, Henry: Corinne Le Quéré: „Could we just adapt to climate change? The answer is no“, in: Financial Times, 18. Juli 2022.

